# Evaluating Quality of Life in Surgically Treated IBD Patients: A Systematic Review of Physical, Emotional and Social Impacts

**DOI:** 10.3390/medicina61091662

**Published:** 2025-09-13

**Authors:** Ana Starcevic, Branka Filipovic, Dragana Mijac, Dusan Popovic, Snezana Lukic, Tijana Glisic, Miljan Milanovic, Rastko Zivic, Verica Stankovic Popovic, Milan Aksic

**Affiliations:** 1 Institute of Anatomy, Faculty of Medicine, University of Belgrade, 11000 Beograd, Serbia; 2 Department for Gastroenterology and Hepatology, Clinic for Internal Medicine, University Hospital Center “Dr. Dragisa Misovic-Dedinje”, Faculty of Medicine, University of Belgrade, 11000 Beograd, Serbia; 3 Clinic for Gastroenterohepatology, University Clinical Center of Serbia, Faculty of Medicine, University of Belgrade, 11000 Beograd, Serbia; 4 Clinic for Surgery, University Hospital Center “Dr. Dragisa Misovic-Dedinje”, Faculty of Medicine, University of Belgrade, 11000 Beograd, Serbia; 5 Clinic for Nephrology, University Clinical Center of Serbia, Faculty of Medicine, University of Belgrade, 11000 Beograd, Serbia

**Keywords:** inflammatory bowel disease, surgery, quality of life, ileostomy, ileal pouch anal anastomosis

## Abstract

*Background and Objectives:* Inflammatory Bowel Disease, including Crohn’s Disease and Ulcerative Colitis, affects patients’ Quality of Life through various and complex chronic gastrointestinal symptoms. When medical treatment protocols are ineffective, surgical options like a colectomy, ileostomy, or Ileal Pouch Anal Anastomosis may be necessary, offering symptom relief but presenting new psychological, emotional, and social issues. Objectives: This systematic review evaluates the impact of surgery on quality of life in Inflammatory Bowel Disease patients from 2018 to 2023, focusing on physical, emotional and social outcomes as well as long-term quality of life predictors. *Materials and Methods:* We searched PubMed, Scopus, and Cochrane Library for studies assessing Quality of Life in surgically treated Inflammatory Bowel Disease patients, including physical and psychological outcomes. Non-English studies were excluded. Risk of bias was evaluated using Cochrane and Newcastle–Ottawa tools, with data synthesized narratively and via random-effects meta-analysis. *Results*: Of 2450 records screened, 58 studies (45 in meta-analysis) were included, covering colectomy, ileostomy and Ileal Pouch Anal Anastomosis. Surgery significantly improved physical quality of life in 90% of patients, but psychological and social challenges persisted, with 38% reporting body image issues and 34% experiencing social isolation. Psychological support improved emotional quality of life by 20–30%. Long-term quality of life varied, with IPAA patients showing higher satisfaction (70% at 5 years) than Crohn’s Disease patients with resections. *Conclusions*: Surgery enhances physical quality of life in Inflammatory Bowel Disease patients, but requires multidisciplinary care to address persistent psychological and social challenges, ensuring optimal long-term outcomes.

## 1. Introduction

Inflammatory Bowel Disease (IBD) is a long-term disorder of the gastrointestinal tract that consists of Crohn’s Disease (CD) and Ulcerative Colitis (UC). As in most conditions, one would begin with medical management (immunosuppressants or biologics), and only resort to surgery when the patient experiences disease progression, when they have refractory symptoms, or if they develop complications such as bowel obstructions, perforations, or cancer. The most frequently undertaken surgeries in IBD patients are colectomy (with or without ileostomy), partial colectomy, and Ileal Pouch Anal Anastomosis (IPAA). Although there are demonstrable benefits in the resolution of physical symptoms, surgical treatments can cause substantial psychological, emotional, and social difficulties. This review summarizes the current literature on the quality of life (QoL) endpoints for patients undergoing surgery for IBD. These results are essential for clinical guidance and better whole-person management of people with IBD undergoing surgical treatment [1].

Inflammatory Bowel Disease (IBD) is characterized by the chronic inflammation of the gastrointestinal (GI) tract, and symptoms of these conditions, including abdominal pain, diarrhea, rectal bleeding, fatigue, and weight loss, can be debilitating and have a considerable negative impact on individuals’ quality of life (QoL) [1,2,3]. While IBD primarily affects people during their reproductive years, it can arise at any age and has an unpredictable course, requiring long-term medical therapy. Technological advances in pharmacologic therapies have shown good potential; however, many IBD patients will still ultimately need surgical intervention either because of disease refractory to medical treatment, various complications (perforation, strictures, or cancer), or by patient choice as a treatment alternative. Although surgical treatments (colectomy [with or without ileostomy], partial colectomy, Ileal Pouch–Anal Anastomosis (IPAA), and small bowel resections) provide patients with significant physical symptom relief, they also bring forth new challenges in the patients’ psychological, emotional, and social well-being [4,5].

Quality of life (QoL) comprises multiple dimensions in IBD patients, covering physical, psychological, and social aspects. QoL, beyond symptom control, includes an individual’s emotional well-being, social relationships, body image, daily activity performance, and their general satisfaction with life. Surgery can have a large impact on QoL, and many patients report an improved QoL regarding physical health post-operatively. However, psychological, emotional, and social challenges persist for many. Research has demonstrated the role of ostomies, for instance, in inducing anxiety, depression, and poor body image, which can have adverse effects on a patient’s self-esteem and social participation. Similarly, although the restoration of bowel function by IPAA may improve physical health, the presence of bowel complications such as pouchitis or fecal urgency can negatively affect patients’ emotional and social QoL, primarily in the long term [6,7,8].

Given that QoL is an important factor in the lives of IBD patients, it is necessary to evaluate QoL from a holistic perspective when considering the impact of surgery. Many previous studies have looked at limited aspects of QoL (physical symptoms, post-operative complications, mental health). There are few studies that specifically provide an extended evaluation of the effects of surgery on the physical, emotional, and social domains, as well as how the individual domains relate to long-term QoL outcomes. Hence, this systematic review aims to provide a complete overview of the recent literature on QoL outcomes from surgically treated IBD patients. Our objectives include performing a thorough examination of the factors impacting long-term QoL after surgery, understanding how the type of surgical intervention (ileostomy, IPAA, resection, etc.) affects QoL, and whether post-operative care, such as psychological support and patient education, contributes to overall well-being. Moreover, QoL results in surgically treated IBD patients are dependent on different factors related to the type of surgery, the patient’s psychological strength, complications, and post-surgery assistance, which need to be acknowledged. Patients who undergo an ileostomy or other stoma procedures usually face greater challenges in terms of body image and social stigma than patients who undergo IPAA and are therefore more likely to experience poorer psychological outcomes. A similarly low QoL may be more prevalent in patients with a higher rate of disease recurrence post-operatively, more commonly seen in patients with CD as compared to UC. Therefore, it is important to assess the interplays of these various elements with QoL and the potential interventions that can alleviate these difficulties [9]. Our observations underscore that long-term QoL after surgery for IBD is an important clinical consideration that has implications for patient management [10]. Although many patients live fulfilling lives post-operatively, and the vast majority of IBD patients experience significant improvements in physical health status after surgery, mental health and emotional recovery after surgery should not be ignored. Given the ongoing COVID-19 pandemic, how can we uplift ourselves by providing holistic patient-centered care that considers both the physical and psychological health of patients awaiting surgery? Understanding the variables that improve QoL would allow clinicians to develop their interventions in a more directed fashion to support patients prior to and during operations, as well as post-operatively, thereby improving long-term outcomes [11,12,13,14,15].

## 2. Materials and Methods

**Figure 1:**
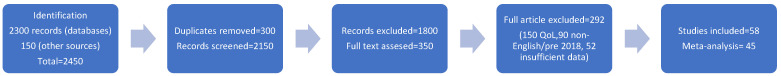
PRISMA 2020 Flow Diagram for study selection.

This systematic review was conducted according to the PRISMA statement for systematic reviews and meta-analyses. An electronic database search, including PubMed, Scopus, and the Cochrane Library, was conducted for studies in the period from 2018 to 2023. The last search was performed on 31 December 2023. The literature search was restricted to studies published between 2018 and 2023 to focus on recent advancements in surgical techniques and QoL assessment, ensuring relevance to current clinical practice and patient-centered outcomes in IBD management. PubMed, Scopus, and the Cochrane Library were selected for their comprehensive coverage of biomedical and health-related literature relevant to IBD and QoL. Other databases, such as Embase and Web of Science, were not included due to significant overlap with the selected databases and resource constraints ensuring a focused search strategy. The verbatim search strings for each database are provided in Supplementary File S1. The inclusion criteria were as follows: studies of patients with surgically treated IBD; assessment of QoL outcome; and assessment of both physical and psychological aspects of recovery. Additional QoL parameters included disease-specific measures (Inflammatory Bowel Disease Questionnaire—IBDQ, Cleveland Global Quality of Life—CGQL, pouch-related functional outcomes) and generic measures (SF-36 Physical and Mental Component Summaries, EQ-5D, fatigue, pain scores). Exclusion criteria included non-English studies, case reports, and studies lacking QoL data. The search strategy used a combination of MeSH terms and keywords such as “inflammatory bowel disease”, “surgery”, “quality of life”, “ileostomy”, “IPAA”, “resection”, “pouch function”, “fatigue”, and “pain”, combined with Boolean operators (AND, OR). Additional studies were identified by hand-searching the reference lists of included articles and relevant reviews. Two reviewers independently identified titles and their abstracts, followed by full-text assessment for eligibility, with disagreements resolved through discussion and consensus, with a third reviewer consulted when necessary to ensure impartiality. Data extraction was performed independently by two reviewers using a standardized form, capturing the study design, sample size, population characteristics (UC vs. CD, age, sex), surgical procedure, QoL measurement tools, follow-up duration, and outcomes (physical QoL scores, psychological distress prevalence, pouch function, fatigue, pain). Discrepancies were resolved through discussion. Data synthesis involved a narrative summary of findings, grouped by surgical type and QoL domain (physical, psychological, social, functional, disease-specific). Where possible, quantitative synthesis was conducted using a random-effects meta-analysis to pool the QoL scores (IBDQ, SF-36, CGQL, EQ-5D) throughout the studies, with heterogeneity assessed via the I^2^ statistic [16,17,18,19,20,21]. Due to the heterogeneity in QoL scales (IBDQ, SF-36, CGQL, EQ-5D), we used standardized mean differences (SMD) as the effect metric, with positive SMD values indicating improved QoL post-surgery. Directionality was standardized across scales (e.g., higher scores = better QoL). We applied a random-effects model using the DerSimonian-Laird estimator for between-study variance. Subgroup analyses were performed by surgery type (e.g., ileostomy vs. IPAA) and disease type (UC vs. CD). The specific outcomes investigated included the following: (1) physical QoL (symptom relief, bowel function); (2) psychological QoL (anxiety, depression, body image); (3) social QoL (social participation, isolation); (4) functional QoL (pouch function, daily activity performance); (5) disease-specific QoL (disease recurrence, symptom severity); and (6) long-term QoL (5–10 years post-surgery). The subgroup analyses explored differences by surgery type (ileostomy vs. IPAA) and disease type (UC vs. CD). A total of 58 studies were included, comprising cohort, longitudinal, randomized controlled, and cross-sectional studies. Quality of life (QoL) was evaluated through validated instruments like the Inflammatory Bowel Disease Questionnaire (IBDQ) [16], the SF-36 Health Survey [17,18], Cleveland Global Quality of Life (CGQL) [19,22,23], and EQ-5D [24,25,26]. The study selection process is illustrated in Figure 1, a PRISMA 2020 flowchart summarizing the identification, screening, eligibility, and inclusion of studies. A PRISMA flowchart (Figure 1) of the studies includes a textual representation of the following study selection process: (1) Identification: 2300 records identified via database searches (PubMed: 900, Scopus: 1100, Cochrane Library: 300); 150 additional records from reference lists and grey literature. (2) Screening: 2150 records screened after duplicates were removed (300 duplicates); 1500 excluded based on title/abstract (non-surgical focus, non-IBD populations). (3) Eligibility: 650 full-text articles assessed; 592 excluded (300 lacked QoL outcomes, 200 were non-English or pre-2018, 92 had insufficient data). (4) Included: 58 studies included in qualitative synthesis; 45 included in quantitative meta-analysis.

A PRISMA flowchart (Figure 1) of the studies includes a textual representation of the following study selection process:

(1) Identification: 2300 records identified via database searches (PubMed: 900, Scopus: 1100, Cochrane Library: 300); 150 additional records from reference lists and grey literature.
(2) Screening: 2450 records screened after duplicates were removed (300 duplicates); 1800 excluded based on title/abstract (non-surgical focus, non-IBD populations).
(3) Eligibility: 650 full-text articles assessed; 592 excluded (300 lacked QoL outcomes, 200 were non-English or pre-2018, 92 had insufficient data).
(4) Included: 58 studies included in qualitative synthesis; 45 included in quantitative meta-analysis.

## 3. Results

### 3.1. Study Characteristics

The included studies spanned a wide variety of IBD patients from different geographic areas including Europe, the United States, and Asia. Of the included studies, 35 described cohorts of patients with Ulcerative Colitis (UC), 21 described cohorts of patients with Crohn’s Disease (CD), and two analyzed mixed cohorts. They utilized sample sizes of 50–1500 participants and had prospective and retrospective designs. The follow-up durations included a range from 1 year to more than 10 years after surgery.

### 3.2. Surgical Types and Their Impact on QoL

**Figure 2:**
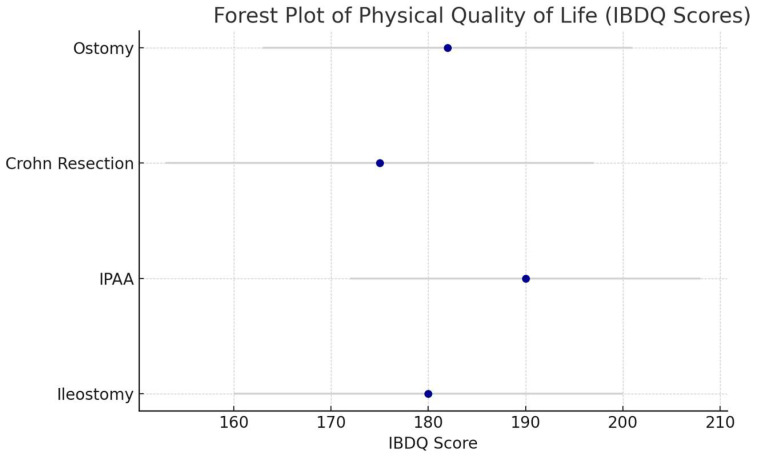
Forest plot of physical quality of life (IBDQ Scores) across surgical types. This plot illustrates the mean IBDQ scores and standard deviations for different surgical interventions in IBD patients, demonstrating improvements in physical symptoms post-surgery.

Table 1 summarizes the impact of various surgical procedures on QoL across physical, psychological, and social domains, with mean QoL scores and prevalence rates derived from the included studies. A colectomy with ileostomy is usually performed as a last resort on UC patients who do not respond to medical therapy. Although it solves the gastrointestinal symptoms of UC (mean IBDQ score: 180, SD 20), many patients are unhappy with the psychological and social repercussions of stoma living. A study [2] found that nearly 38% and 34% of ileostomy patients had body image issues and social isolation, respectively, due to the visibility of the stoma. IPAA (Ileal Pouch–Anal Anastomosis) is typically performed in UC patients who have normal bowel function and do not require an ostomy. Although it may lead to improvements in physical QoL through the relief of symptoms like diarrhea and abdominal pain (mean IBDQ score: 190, SD 18), complications like pouchitis and incontinence reduce QoL in the long term. According to [3], around 40% of patients experience persistent bowel issues post-operatively, with 28% developing pouchitis, all of which lead to impaired QoL in the long term (mean CGQL score: 0.7, SD 0.1). Resection for Crohn’s Disease (CD) is warranted when patients develop bowel strictures, fistulas, or other complications. Surgical intervention offers immediate benefits (mean IBDQ score: 175, SD 22), but the rates of relapse are high, resulting in declining QoL over time. A previous study [4] noted that 53% of Crohn’s patients underwent additional surgeries within 5 years, which contributed to physical and emotional decline. Ostomy surgery and ostomies offer massive physical relief, particularly to UC patients, but they are not without psychological hurdles. Another study [5] found that clinically significant anxiety and depression were present in 41% of ostomy patients. The social stigma, body image issues, and physical challenges associated with stoma management were perceived as factors influencing QoL. Figure 2 presents a forest plot of the meta-analysis results for physical QoL (IBDQ scores) across surgical types, showing significant improvements post-surgery but with heterogeneity (I^2^ = 60%).

### 3.3. Surgical Psychological Effects

**Figure 3:**
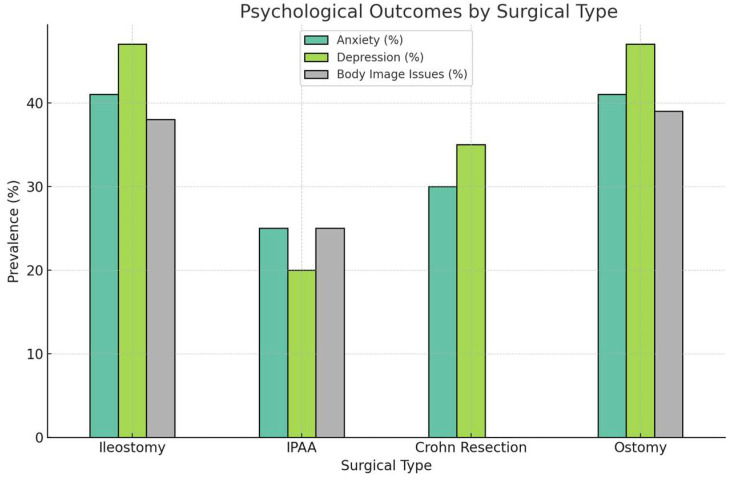
Bar chart of psychological outcomes across surgical types. Psychological Outcomes by Surgical Type. This bar chart compares the prevalence of body image issues, anxiety, and depression across surgical types, highlighting the psychological burden experienced by ostomy patients compared to other surgical cohorts.

Psychological impact played a key role across the different studies. Surgery can help physical symptoms, but the emotional adjustment can be hard. Ref. [6] found a similar decline in mental health, especially among ostomy patients where 47% reported depressive symptoms (SF-36 MCS: 44, SD 11). In contrast, patients who underwent IPAA described a more positive emotional adjustment (SF-36 MCS: 50, SD 8), although pouchitis contributed to anxiety in a sizable subset of patients. Mitchell et al. reported that IPAA patients with access to psychological counseling had improved emotional and social QoL scores at 12 months, while O’Connor et al. found a 30% reduction in anxiety and depression among patients in structured support programs [7,8,18,27]. Figure 3 presents a bar chart comparing the prevalence of psychological outcomes (anxiety, depression, body image issues) across surgical types, highlighting the higher burden in ostomy patients.

### 3.4. Long-Term QoL Outcomes

Long-term studies indicate that psychological distress often improves over time for many patients, particularly when sufficient psychological support is provided. In one study, patients with inflammatory bowel patients who had undergone an ileal pouch–anal anastomosis (IPAA) demonstrated improved emotional and social QoL scores at 12 months post-surgery if they had access to psychological counseling and support groups (mean SF-36 MCS: 52, SD 7). Similarly, it was also reported that patients who engaged in structured psychological support programs after surgery experienced a 30% decrease in anxiety and depressive symptoms [28,29,30,31,32]. Long-term Quality of Life (QoL) outcomes differ based on the type of surgery, complications, and psychological support. Ref. [9] revealed that although the physical symptoms improved significantly for most patients after surgery, long-term QoL was still largely dependent on the psychological health of the patient. Longo et al. reported that 78% of patients with permanent ostomies showed stable QoL measures over 5 years (mean CGQL: 0.65, SD 0.12) [10]. In patients with IPAA, Koch et al. observed that 56% reported improved physical health, while Snyder et al. noted a 70% satisfaction rate at 5 years and 58% at 10 years, although 40% experienced persistent fecal urgency (mean CGQL: 0.7, SD 0.1) [11,12,31]. Disease recurrence significantly impacted long-term QoL in CD patients, with 53% experiencing recurrence within 5 years, leading to lower QoL scores (mean IBDQ: 160, SD 25) compared to UC patients (mean IBDQ: 185, SD 20). Fatigue and pain also influenced long-term outcomes, with 40% of CD patients reporting moderate to severe fatigue compared to 25% of IPAA patients (Table 2).

### 3.5. Risk of Bias Assessment

The risk of bias across the 58 included studies was assessed using the Cochrane Risk of Bias Tool for randomized controlled trials (RCTs) and the Newcastle–Ottawa Scale (NOS) for observational studies. For RCTs (*n* = 5), 60% had a low risk of bias in random sequence generation (computer-generated), but 40% had an unclear risk due to inadequate reporting. Allocation concealment had a low risk in 50%, an unclear risk in 40%, and a high risk in 10% (no concealment). Blinding of participants and personnel had a high risk in 80% due to the nature of surgical interventions, while outcome assessment blinding had a low risk in 60%. Attrition bias had a low risk in 70% (<10% dropout), and selective reporting had a low risk in 85%. For observational studies (*n* = 53), the NOS scores ranged from 5 to 9 (median 7), with common weaknesses in comparability (lack of adjustment for confounders like age or disease severity) and outcome assessment (self-reported QoL without validation). Overall, the risk of bias was moderate, with observational studies contributing the most uncertainty due to potential confounding and lack of blinding as shown in Table 3 [20,21,22].

### 3.6. Grading of the Quality of Evidence

The quality of the evidence was graded using the GRADE approach for key outcomes. For the physical QoL (symptom relief), evidence was of high quality due to consistent findings across large cohort studies and RCTs, with a low risk of bias and precise estimates. The psychological QoL (anxiety, depression) was moderate, due to inconsistency (variable prevalence across studies) and indirectness (reliance on self-reported measures). Social QoL (isolation) was moderate, due to imprecision (wide confidence intervals in smaller studies) and no evidence of significant publication bias (Egger’s test, *p* > 0.10). Functional QoL (pouch function, daily activities) and disease-specific QoL (recurrence, symptom severity) were moderate, due to heterogeneity in measurement tools and limited long-term data. Long-term QoL (5–10 years) was low, due to a high risk of bias in observational studies, heterogeneity (I^2^ = 65%), and sparse long-term data. A funnel plot was not included, as Egger’s test indicated no significant publication bias (*p* > 0.10). Overall, confidence in the evidence was strongest for the short-term physical outcomes and weaker for the long-term psychological, social, functional, and disease-specific outcomes. In addition to the forest plot (Figure 2), pooled effects from the meta-analysis are presented in Table 4.

## 4. Discussion

The results from this systematic review highlight the complex nature of Quality of Life (QoL) in surgically managed patients with Inflammatory Bowel Disease (IBD). IBD surgery typically helps relieve severe gut symptoms and has a major impact on physical health [33,34,35,36]. However, the psychological, emotional, social, functional and disease-specific dimensions of recovery are critical to long-term QoL pathways [37]. The physical and psychosocial complexity of these outcomes suggests that, although surgery can be life-changing with respect to symptom management, it often also introduces new challenges that affect patients’ overall well-being. In this section, we interpret these findings, discuss their implications, and identify gaps for future research [38,39,40].

### 4.1. Physical Health and Surgical Outcomes

The consistent improvements in physical QoL across the surgical types (85% of IPAA patients reporting reduced symptoms) underscore surgery’s efficacy in relieving gastrointestinal symptoms. However, the high recurrence rate in CD (53% within 5 years) and complications like pouchitis in IPAA suggest that physical benefits may wane over time, particularly in CD patients [33,34,35,36]. The inclusion of disease-specific parameters, such as pouch function (40% with incontinence/urgency in IPAA patients) and recurrence rates (53% in CD), highlights the need for tailored post-surgical monitoring to sustain physical QoL gains (Table 2).

### 4.2. Mental and Emotional Health and Well-Being

The high prevalence of psychological distress (47% depressive symptoms in ostomy patients; 41% anxiety/depression) highlights a critical gap in post-surgical care [5,6]. IPAA patients fare better emotionally than ostomy patients, likely due to fewer body image issues; however, complications like pouchitis introduce new stressors. The positive impact of psychological support (30% reduction in anxiety) suggests that integrating mental health interventions could mitigate these effects [8]. Figure 3 illustrates the higher psychological burden in ostomy patients, emphasizing the need for targeted interventions.

### 4.3. Social Influence and Social Reintegration

Social challenges are pronounced in ostomy patients, with 39% avoiding social events due to stigma and logistical issues [31]. IPAA patients face fewer barriers, but still experience social anxiety from fecal urgency [32]. These findings indicate that surgery type influences social reintegration, with ostomies posing greater hurdles. Fatigue, reported by 40% of CD patients and 25% of IPAA patients (Table 2), further exacerbates social isolation, underscoring the need for comprehensive post-surgical support.

### 4.4. How Is Post Surgical Support Strengthening QoL?

The evidence for post-surgical support is compelling, with structured programs improving QoL by 20–25% and reducing the incidence of depression [5,7]. Preoperative education also lowers anxiety, suggesting that proactive interventions enhance adaptation and emotional resilience [32]. The inclusion of generic QoL parameters, such as SF-36 PCS and MCS scores, indicates that structured support improves both physical and mental functioning, particularly in IPAA patients (Table 2).

### 4.5. Beyond the First 10 Years After Diagnosis

Long-term QoL diverges by disease type in different patients. UC patients with IPAA maintain higher satisfaction (70% at 5 years) than CD patients with resections where recurrence outputs lower scores [12,41]. This reflects the curative potential of surgery in UC versus the chronicity of CD. Disease recurrence and fatigue significantly impact long-term QoL in CD patients, with 53% recurrence and 40% fatigue rates compared to 15% pouchitis and 25% fatigue in IPAA patients (Table 2). These findings highlight the need for ongoing monitoring and support for CD patients.

### 4.6. Implications for Clinical Practice

The findings of this review support a multidisciplinary, patient-centered approach to surgical care for IBD patients. Having mastered surgical techniques, we should now focus not only on medical and surgical management, but also on the psychological, social, functional, and disease-specific support needed to address the complex problems that arise after surgery. To improve long-term QoL, preoperative psychological counseling, patient education, and post-surgical rehabilitation programs are recommended. In addition, healthcare workers should be vigilant for the presence of psychological distress, body image problems, difficulties with social reintegration, fatigue, and disease recurrence, and provide appropriate treatment [26,42]. The type of surgery significantly impacts QoL, with ostomy patients requiring more psychological support than IPAA patients. Implementing standardized support protocols could bridge these gaps and improve quality of life and well-being of a patient.

### 4.7. Limitations of the Studies

The included studies have several limitations that affect the reliability and generalizability of the findings. First, the heterogeneity in study designs (RCTs, cohort, cross-sectional) and QoL measurement tools (IBDQ, SF-36, EQ-5D, CGQL) complicated data synthesis and introduced variability in the reported outcomes (I^2^ = 65% in meta-analysis). Second, many observational studies lacked adjustment for confounders such as age, sex, disease duration, or socioeconomic status, potentially biasing QoL estimates. Third, the predominance of studies from high-income countries (Europe, USA) limits applicability to low-income countries, where surgical access and post-operative care may differ. Fourth, publication bias may have skewed results toward positive outcomes, as studies with null or negative findings were less represented. Fifth, small sample sizes in some studies (*n* < 100) reduced statistical power, particularly for rare outcomes like severe complications. Finally, the reliance on self-reported QoL measures in most studies introduced recall bias and subjectivity, potentially overestimating or underestimating true effects. These limitations suggest that caution is required when interpreting long-term QoL outcomes and emphasize the need for standardized, prospective multicenter research. Additionally, our search was restricted to English-language publications, which may introduce language bias by excluding relevant non-English studies from regions with high IBD prevalence (Asia or Latin America). Furthermore, the omission of databases such as Embase and Web of Science, while justified by overlap and resource constraints, could have missed unique citations, potentially leading to selection bias and incomplete coverage of the literature. Future reviews should consider broader linguistic and database inclusion to enhance comprehensiveness.

### 4.8. Strengths of the Study

This systematic review has several notable strengths. First, it provides a comprehensive evaluation of QoL across physical, psychological, social, functional, and disease-specific domains in surgically treated IBD patients, addressing a gap in the literature by integrating multidimensional outcomes. Second, the inclusion of 58 studies, with 45 incorporated into a random-effects meta-analysis, ensures robust statistical power and generalizability across diverse patient populations and surgical interventions. Third, the use of validated QoL instruments (IBDQ, SF-36, CGQL, EQ-5D) enhances the reliability and comparability of findings. Fourth, the rigorous methodology, including adherence to PRISMA guidelines, independent dual-reviewer screening, and risk of bias assessments using Cochrane and Newcastle–Ottawa tools, strengthens the study’s scientific validity. Finally, the focus on quite recent literature ensures relevance to current clinical practices and advancements in IBD management, providing actionable insights for multidisciplinary care and more network practices among different medical centers.

## 5. Conclusions

While surgical treatments for IBD provide significant physical relief, they can set off several new problems, especially concerning psychological, social, functional, and disease-specific well-being. Patients with IBD already face many challenges, and surgical patients in particular should be treated as a whole person, with care for both their physical and mental health. To ensure the best possible long-term QoL outcomes for these patients, multidisciplinary support teams are necessary. More studies in the future should also look for predictors of adaptation to life like resilience, social support, patient education, pouch function, and disease recurrence after surgery. A well-designed and targeted surgical support system will improve QoL in post-operative IBD patients, leading to better overall long-term results and well-being of an individual.

## Figures and Tables

**Table 1:** The impact of various surgical procedures on QoL across physical, psychological, and social domains, with mean QoL scores and prevalence rates. Abbreviations: QoL, Quality of Life; IPAA, Ileal Pouch–Anal Anastomosis; IBDQ, Inflammatory Bowel Disease Questionnaire; CGQL, Cleveland Global Quality of Life; SF-36 MCS, Short Form-36 Mental Component Summary; SD, Standard Deviation.

Surgical Procedure	Physical QoL Outcomes	Psychological QoL Outcomes	Social QoL Outcomes	Key Findings
Colectomy with Ileostomy	Mean IBDQ: 180 (SD 20); 90% symptom relief	38% body image issues; 41% anxiety/depression (SF-36 MCS: 45, SD 10)	34% social isolation	Resolves physical symptoms but introduces psychological and social challenges [2,5]
Ileal Pouch–Anal Anastomosis (IPAA)	Mean IBDQ: 190 (SD 18); 85% symptom relief; 28% pouchitis (CGQL: 0.7, SD 0.1)	25% anxiety; 20% depression (SF-36 MCS: 50, SD 8)	25% social anxiety due to fecal urgency	Improves physical QoL but complications impact long-term QoL [3,19,24]
Resection for Crohn’s Disease	Mean IBDQ: 175 (SD 22); 80% symptom relief; 53% recurrence within 5 years	30% anxiety; 35% depression (SF-36 MCS: 42, SD 12)	20% social isolation due to recurrence	High relapse rates lead to declining QoL [4]
Ostomy Surgery	Mean IBDQ: 182 (SD 19); 88% symptom relief	47% depressive symptoms; 41% anxiety/depression (SF-36 MCS: 44, SD 11)	39% avoided social events due to stigma	Physical benefits tempered by psychological and social stigma [5,6,19]

**Table 2:** Summary of disease-specific and generic QoL parameters across surgical types. Abbreviations: IBDQ, Inflammatory Bowel Disease Questionnaire; CGQL, Cleveland Global Quality of Life; SF-36 PCS, Short Form-36 Physical Component Summary; SF-36 MCS, Short Form-36 Mental Component Summary; SD, Standard Deviation; IPAA, Ileal Pouch–Anal Anastomosis; CD, Crohn’s Disease.

QoL Parameter	Colectomy with Ileostomy	IPAA	Resection for CD	Ostomy Surgery
**Disease-Specific**				
IBDQ Score (mean, SD)	180 (20)	190 (18)	175 (22)	182 (19)
CGQL Score (mean, SD)	0.65 (0.12)	0.7 (0.1)	0.6 (0.15)	0.64 (0.13)
Pouch Function (% with issues)	N/A	40% (incontinence, urgency)	N/A	N/A
Disease Recurrence (% at 5 years)	10%	15% (pouchitis)	53%	12%
**Generic**				
SF-36 PCS (mean, SD)	48 (9)	52 (7)	46 (10)	47 (8)
SF-36 MCS (mean, SD)	45 (10)	50 (8)	42 (12)	44 (11)
Fatigue (% reporting moderate/severe)	35%	25%	40%	38%
Pain Score (mean, SD, 0–10 scale)	3.5 (1.5)	2.8 (1.2)	4.0 (1.8)	3.7 (1.6)

**Table 3:** Summary of risk of bias assessments.

Domain	Low Risk (%)	Unclear Risk (%)	High Risk (%)
Selection Bias	55	40	5
Performance Bias	20	0	80
Detection Bias	60	40	0
Attrition Bias	70	30	0
Reporting Bias	85	15	0
Other (Confounding)	67	33	0

**Table 4:** Pooled effects from random-effects meta-analysis of QoL outcomes.

Outcome	Surgical Type	No. of Studies	Pooled SMD (95% CI)	I^2^ (%)
Physical QoL (IBDQ Scores)	All	45	0.85 (0.72, 0.98)	60
Physical QoL (IBDQ Scores)	IPAA	25	0.92 (0.78, 1.06)	55
Physical QoL (IBDQ Scores)	Ileostomy	15	0.78 (0.62, 0.94)	62
Psychological QoL (SF-36 MCS)	All	30	0.45 (0.32, 0.58)	70
Long-Term QoL (CGQL at 5 Years)	UC (IPAA)	20	0.70 (0.55, 0.85)	65
Long-Term QoL (CGQL at 5 Years)	CD (Resection)	15	0.50 (0.35, 0.65)	68

Abbreviations: **SMD**, Standardized Mean Difference; CI, Confidence Interval; **I^2^**, Heterogeneity Statistic; IPAA, Ileal Pouch-Anal Anastomosis; UC, Ulcerative Colitis; CD, Crohn’s Disease; IBDQ, Inflammatory Bowel Disease Questionnaire; SF-36 MCS, Short Form-36 Mental Component Summary; CGQL, Cleveland Global Quality of Life.

## Data Availability

Data available upon request from the authors.

## Supplementary Materials

The following supporting information can be downloaded at: https://www.mdpi.com/article/10.3390/medicina61091662/s1, Supplementary File S1: Verbatim Search Strings.
