# Supplementary material for: Evaluating Quality of Life in Surgically Treated IBD Patients: A Systematic Review of Physical, Emotional and Social Impacts
Source: Medicina (Kaunas). 2025 Sep 13;61(9):1662. doi: 10.3390/medicina61091662 (PMC12471534; doi:10.3390/medicina61091662)
Supplement: Supplementary file 1 [file medicina-61-01662-s001.zip › medicina-3681180-supplementary.pdf]

medicina

MDPI

Supplementary Materials: Supplementary File S1: Verbatim Search Strings

PubMed: ("inflammatory bowel disease"[MeSH Terms] OR "inflammatory bowel diseases"[All Fields] OR IBD[All Fields] OR "crohn disease"[MeSH Terms] OR "crohn's disease"[All Fields] OR "ulcerative colitis"[MeSH Terms] OR "colitis, ulcerative"[All Fields]) AND (surgery[MeSH Terms] OR surgical[All Fields] OR colectomy[All Fields] OR ileostomy[All Fields] OR "ileal pouch anal anastomosis"[All Fields] OR IPAA[All Fields] OR resection[All Fields]) AND ("quality of life"[MeSH Terms] OR QoL[All Fields] OR "pouch function"[All Fields] OR fatigue[All Fields] OR pain[All Fields]) AND ("2018/01/01"[PDAT] : "2023/12/31"[PDAT])

Scopus: (TITLE-ABS-KEY ("inflammatory bowel disease" OR IBD OR "Crohn's Disease" OR "Ulcerative Colitis") AND TITLE-ABS-KEY (surgery OR colectomy OR ileostomy OR "Ileal Pouch Anal Anastomosis" OR IPAA OR resection) AND TITLE-ABS-KEY ("quality of life" OR QoL OR "pouch function" OR fatigue OR pain)) AND PUBYEAR > 2017 AND PUBYEAR < 2024

Cochrane Library: [inflammatory bowel disease OR IBD OR Crohn OR ulcerative colitis] in Title Abstract Keyword AND [surgery OR colectomy OR ileostomy OR IPAA OR resection] in Title Abstract Keyword AND [quality of life OR QoL OR pouch function OR fatigue OR pain] in Title Abstract Keyword (Word variations have been searched) Publication Year from 2018 to 2023
